# Maternal serum glycosylated fibronectin as a short-term predictor of preeclampsia: a prospective cohort study

**DOI:** 10.1186/s12884-020-2809-2

**Published:** 2020-02-24

**Authors:** Evelyn A. Huhn, Ina Hoffmann, Begoña Martinez De Tejada, Soeren Lange, Kylie M. Sage, Charles T. Roberts, Michael G. Gravett, Srinivasa R. Nagalla, Olav Lapaire

**Affiliations:** 1 Department of Obstetrics and Gynaecology, University Hospital Basel, Basel, Switzerland; 2 Department of Obstetrics and Gynaecology, Faculty of Medicine, Geneva University Hospitals, Geneva, Switzerland; 3 Department of Obstetrics and Gynecology, Institutions Hospital du Nord Vaudois, Yverdon-les-Bains, Switzerland; 4 DiabetOmics, Inc, Hillsboro, OR 97006 USA; 5 Biostatistics and Design Program, School of Public Health, Oregon Health & Science University, Portland, OR 97239 USA; 6 Department of Obstetrics and Gynecology, University of Washington Medical Center, Seattle, WA 98195 USA

## Abstract

**Background:**

Preeclampsia is a major pregnancy complication that results in significant maternal and infant mortality, most of which occurs in low and middle-income countries. The accurate and timely diagnosis of preeclampsia is critical in management of affected pregnancies to reduce maternal and fetal/neonatal morbidity and mortality, yet difficulties remain in establishing the rigorous diagnosis of preeclampsia based on clinical parameters alone. Biomarkers that detect biochemical disease have been proposed as complements or alternatives to clinical criteria to improve diagnostic accuracy. This cohort study assessed the performance of several biomarkers, including glycosylated fibronectin (GlyFn), to rule-in or rule-out preeclampsia within 4 weeks in a cohort of women at increased risk for preeclampsia.

**Methods:**

151 women with risk factors for or clinical signs and symptoms of preeclampsia were selected from a prospective cohort. Maternal serum samples were collected between 20 and 37 weeks of gestation. Clinical suspicion of preeclampsia was defined as presence of new-onset proteinuria, or clinical symptoms of preeclampsia. Subjects with a clinical diagnosis of preeclampsia at the time of enrollment were excluded. GlyFn, pregnancy-associated plasma protein-A2 (PAPPA2), placental growth factor (PlGF), and soluble fms-like tyrosine kinase-1 (sFlt-1) were measured by immunoassay. GlyFn was also determined using a rapid point-of care (POC) test format. Receiver-operating characteristic (ROC) curves derived from logistic regression analysis were used to determine the classification performance for each analyte.

**Results:**

32 of 151 (21%) women developed a clinical diagnosis of preeclampsia within 4 weeks. All biomarkers exhibited good classification performance [GlyFn (area under the curve (AUROC) = 0.94, 91% sensitivity, 86% specificity); PAPPA2 AUC = 0.92, 87% sensitivity, 77% specificity; PlGF AUC = 0.90, 81% sensitivity, 83% specificity; sFlt-1 AUC = 0.92, 84% sensitivity, 91% specificity. The GlyFn immunoassay and the rapid POC test showed a correlation of r = 0.966.

**Conclusions:**

In this prospective cohort, serum biomarkers of biochemical disease were effective in short-term prediction of preeclampsia, and the performance of GlyFn in particular as a POC test may meet the needs of rapid and accurate triage and intervention.

## Background

Preeclampsia (PE) is associated with 10–15% of all maternal deaths during pregnancy and childbirth, making it the second-leading cause of maternal mortality, resulting in an estimated 76,000 maternal deaths annually [1–3]. PE also accounts for 25% of stillbirths and 25% of neonatal deaths [4]. Over 99% of this maternal and fetal/neonatal mortality attributed to PE occurs in low-and middle-income countries, in particular Africa and the Indian subcontinent [5]. Previous studies suggest that mortality rates could be considerably reduced if clinicians were more aware of the likelihood that PE could develop [6, 7]. PE was redefined by the American College of Obstetricians and Gynaecologists (ACOG) in 2013 [8]. Specifically, the “traditional” diagnostic criteria of new-onset hypertension > 140/90 mmHg and proteinuria > 300 mg/24 h after 20 weeks of gestation were revised, and proteinuria is no longer required as long as other maternal organ dysfunction (i.e., renal insufficiency, liver involvement, neurological and hematological complications) is present. The International Society for the Study of Hypertension in Pregnancy (ISSHP), the Australasian Society for the Study of Hypertension in Pregnancy, and the Society of Obstetricians and Gynaecologists of Canada added utero-placental dysfunction or intrauterine growth restriction (IUGR) to the diagnostic criteria for PE [9–11].

Identification of the clinical features consistently associated with PE is further complicated by the existence of cases of PE with the same underlying placental pathology, but that exhibit no signs of hypertension [12]. Eclampsia and the syndrome of Hemolysis, Elevated Liver enzymes, and Low Platelets (HELLP) can also occur in the absence of hypertension or proteinuria [13]. These “non-traditional” constellations of symptoms contribute to the difficulty in obtaining an accurate diagnosis of PE solely based on clinical criteria. This is particularly problematic in women with pre-existing proteinuria and pre-existing or gestational hypertension, in whom accurate diagnosis of PE is critical. More objective measures to help clinicians make a final and accurate diagnosis would greatly improve clinical care and in many cases could be lifesaving.

An important alternative to diagnoses based on observable clinical presentation is the determination of the levels of predictive biomarkers that can be measured in body fluids such as blood, urine, or saliva. A number of circulating factors have been shown to be associated with PE, including soluble endoglin, placental growth factor (PlGF), soluble fms-like tyrosine kinase-1 (sFlt-1), vascular endothelial growth factor (VEGF), pregnancy-associated plasma protein A-2 (PAPPA2), glycosylated fibronectin (GlyFn), vasopressin, and copeptin [14–18].

In this study, we evaluated the ability of several of the biomarkers GlyFn, PAPPA2, PlGF, and sFlt-1, to predict the development of PE within 40 days of maternal sampling. The hypothesis is that GlyFn and PAPPA2 have comparable test performance as the known biomarkers PlGF and sFlt-1. We also describe a point-of-care (POC) test for GlyFn (Lumella™) and determine its test performance in comparison to the standard GlyFn immunoassay.

## Methods

### Study design and patients

We present a prospective, observational study which was conducted at the University Hospitals in Basel and Geneva, Switzerland [19]. The Competent Ethics Committee of Northwestern Switzerland and Geneva (IRB approval numbers EKNZ PB_2016_02490 and GE 14–216) approved the study protocol, and written informed consent was obtained from all participants. Women who were > 18 years of age with a singleton pregnancy were included if they had at least one PE risk factor: nulliparous overweight or obese women with body mass index (BMI) > 26.1 kg/m^2^, nulliparous women > 40 years of age, pre-existing diabetes, essential hypertension or renal disease, pregnancy-induced hypertension, gestational diabetes (defined by at least one pathological value of fasting glucose (> 5.1 mmol/l) or at one (> 10.0 mmol/l) or two hours (> 8.5 mmol/l) after a 75-g glucose load, utero-placental dysfunction (defined by abnormal uterine perfusion with mean pulsatility index >95th percentile in the second trimester and/or bilateral uterine artery notching), previous PE, eclampsia, or HELLP, thrombophilia with high risk for PE (homozygous factor V Leiden or methylenetetrahydrofolate reductase (MTHFR) C677T defects, or the combination of heterozygous factor II G20210A and heterozygous factor V Leiden defects diagnosed in a DNA analysis prior pregnancy), antiphospholipid antibodies, or family history of PE, eclampsia, or HELLP in first-degree relatives. Additionally, women who had symptoms suspicious of PE (two combined findings of clinical symptoms like headache and/or scotoma and/or epigastric pain and/or excessive edema and/or new onset proteinuria (> 1+ in dipstick)) were asked to participate. Exclusion criteria included diagnosis of PE at sample collection, chromosomal aberrations, fetal malformations, abortion, or stillbirth at < 22 weeks of gestation. All eligible women were followed regularly with recording of demographic characteristics, medical history, clinical examinations, and blood draws for biomarker analysis (GlyFn, PAPPA2, PlGF, and sFlt-1). High-risk women with suggestive clinical findings and symptomatic women were treated expectantly, depending on their clinical condition, until delivery. The results of the biomarker analysis were not available until the end of study and did not, therefore, influence management decisions.

### Diagnostic criteria for hypertensive diseases in pregnancy

Pre-existing hypertension was defined as systolic blood pressure > 140 mmHg and/or diastolic blood pressure > 90 mmHg diagnosed before conception or < 20 weeks of gestation. Gestational hypertension was determined as new onset of hypertension developing > 20 weeks of gestation without proteinuria. The following criteria for PE were used to establish the diagnosis: New-onset systolic blood pressure > 140 mmHg and/or diastolic blood pressure > 90 mmHg measured on two occasions at least 6 h apart but within one week and new-onset proteinuria with > 30 mg/24-h urine protein collection or > 2+ in dipstick or spot urine (> 3 mg/dL or protein/creatinine ratio > 3 mg protein/mmol creatinine) > 20 weeks of gestation. Eclampsia was defined as new onset of tonic-clonic seizures associated with PE, which could not be assigned to any other cause. HELLP syndrome was considered when haemolysis (lactic acid dehydrogenase > 600 IU/L, and/or lowered haptoglobin), elevated liver enzymes (aspartate amino transferase exceeding 70 IU/L) and low platelets (platelet counts < 100,000/μL) occurred.

### Diagnostic criteria for intrauterine growth restriction (IUGR)

IUGR was defined as an estimated fetal weight < 10th percentile (adjusted for gender and ethnicity according to charts routinely used by both sites [20]) plus pathological finding(s) in Doppler indices (cerebro-placental ratio < 5th percentile and/or a uterine artery pulsatility index >95th percentile in the second trimester) or a birth weight < 3rd percentile [21].

### Assessment of GlyFn, PAPPA2, PlGF, and sFlt-1

All maternal serum samples were aliquoted and stored at − 80 °C until analysis. Commercial immunoassay kits for sFlt-1 and PlGF (R&D systems, Minneapolis, MN, USA), PAPPA2 (Ansh Labs, Webster, TX, USA), and GlyFn (DiabetOmics, Inc., Hillsboro, OR, USA) were used according to manufacturer’s instructions. Inter-assay coefficients of variation for these commercial kits ranged from 1.89–6.65% and the intra-assay coefficients ranged from 2.1–4.5%. Biomarker thresholds for PlGF and sFlt-1 were chosen based on published literature using R&D immunoassays [22]; abnormal PlGF levels are those < 100 pg/ml and abnormal sFlt-1 levels are those > 7000 ng/ml. The threshold for PAPPA2 > 200 ng/ml was determined from prior biomarker studies (unpublished data). GlyFn threshold > 315 μg/ml were derived from the current dataset which best discriminated cases from non-cases and require additional validation in future studies.

### Point-of-care test (Lumella™ test system)

A prototype GlyFn POC test strip was previously described that employed a fluorescently labeled fibronectin polyclonal antibody as both the detection and capture antibody, with the signal from maternal serum measured using a commercial automated cassette reader [18]. In the current study, serum samples were analyzed for GlyFn using the second-generation Lumella™ PE test (DiabetOmics, Inc.) according to the manufacturer’s instructions. Test strips were configured with monoclonal antibodies against GlyFn labeled with gold particles for quantification using a hand-held Lumella™ reader system. Briefly, 5 μl of serum is diluted 1:350 in running buffer and 120 μl of diluted serum is added to the test strip and inserted into the reader. The GlyFn concentration is displayed on the reader at the end of 10 min. Calibration information is supplied by the manufacturer as a lot-specific radiofrequency identification (RFID) tag on each test kit. The measurable range of the Lumella™ assay is 100 ng/mL to 800 μg/mL vs 10–2000 μg/mL for the prototype version [16]. The intra-/interassay coefficients of variation at mean concentrations of 50–800 μg/mL were 8.6/10.4 and 9.2/10.2%, respectively.

### Participant/sample selection

From a prospective cohort, 226 unique samples were collected. Fifty-seven samples were excluded as we restricted the current investigation to samples derived > 20 and < 37 weeks of gestation and to women who developed clinical PE within 40 days of sample collection or did not develop PE but had a sample collected at similar gestational age. High-risk samples were chosen based on matching for gestational age (within 1 week). No exclusions were needed because of the matching of high-risk women. Women with a diagnosis of PE prior to sample collection were excluded from the analysis. Analyses were restricted to one sample per woman and the earliest sample were chosen from women in the PE group who had multiple samples collected to better represent early prediction. Thereby another 18 samples were excluded because of multiple measurement within 40 days period. Finally, 151 women with samples were included in the current analysis.

### Statistical analyses

Baseline maternal characteristics were stratified for women within these groups. The nonparametric, two-sided Wilcoxon rank sum test was used to compare differences between groups for continuous variables, as they are more robust than non-normal distributions, as well as outlying observations. The χ^2^ test was used for categorical variables. We also compared co-morbidities, pre-existing renal disease, pre-existing diabetes, pre-existing hypertension, and gestational hypertension. Biomarker distributions for women with and without development of clinical PE were calculated and compared, and medians and interquartile ranges (IQR) of the original scales are reported. Non-parametric test equivalent to receiver-operating characteristic (ROC) curve were used to inferentially compare biomarker distributions. Confirmed delivery outcomes were also compared between groups, including gestational age at delivery, neonatal birth weight, Apgar scores, cesarean sections, preterm births, IUGR, and SGA.

ROC curves, the area under the curve (AUC), along with corresponding 95% confidence intervals (CIs) for PE diagnosis were generated using predicted probabilities from simple logistic regression models [23]. We estimated and compared the operating characteristics (sensitivity, specificity) using thresholds described previously (> 315 U/mL for GlyFn, > 200 ng/mL for PAPPA-2, < 100 pg/mL for PlGF, and ≥ 7000 ng/mL for sFlt-1) for detection of PE. We evaluated the ability of the various biomarkers to predict the onset of PE within four weeks of sample collection. Predicted probabilities from simple logistic regression were used to create ROC curves, AUCs, and 95% CI’s [23]. A comparison of the GlyFn plate immunoassay to the GlyFn POC test was performed on samples assayed by both methods. Pearson correlation coefficient was calculated to compare the methods. ROC curves were generated for each method to ascertain classification accuracy. All statistical analyses were performed using R (3.2.2) via Rstudio software version 1.0.136 (https://www.rstudio.com/products/RStudio/). ROC curves were created using the pROC package [24].

## Results

### Baseline characteristics

Between September 2011 and July 2015, a total of 151 women meeting the inclusion criteria were enrolled in the final study, 32 (21%) of whom received a clinical diagnosis of PE in 4 weeks from sample collection. The maternal and pregnancy characteristics of both groups are summarized in Table 1. The PE group had a shorter interval between blood sampling and delivery (PE 8 d (±9.7 d) vs. without PE 60 d (±42.9 d), *P* < 0.0001) and delivered earlier in comparison to the without PE group (PE at 31 weeks of gestation (±4.6) vs. without PE at 37 weeks of gestation (±3.5), P < 0.0001). Both groups had notable differences in pregnancy outcome parameters, with lower Apgar scores, lower neonatal birth weight, and higher preterm and higher IUGR rates in the PE group (Table 1).

**Table 1:** Clinical characteristics of the study groups

	At-risk women without PE	At-risk women with PE	*p*-value
*n*	119	32	
*Maternal Characteristics*			
Gestational age at sample collection (wk)	29 (8.5)	30 (7.3)	0.14
Maternal age (yr)	33 (7.5)	33 (6.5)	0.74
Maternal weight pre-pregnancy (kg)	68 (23.8)	65.5 (16.5.)	0.7
Maternal weight at sample collection (kg)	78 (22.8)	80 (15.5)	0.71
Maternal BMI (kg/m^2^)	24.16 (7.8)	25.67 (5.5)	0.96
Systolic BP (mmHg)	124 (19)	160 (39)	< 0.001
Days before delivery	54 (72)	4 (10.8)	< 0.001
Ethnicity: Caucasian	100 (84%)	23 (72%)	0.08
Nulliparity	64 (54%)	20 (63%)	0.5
*Co-morbidities*			
Pre-existing chronic renal disease	7 (6%)	3 (9%)	0.69
Pre-existing diabetes	4 (3%)	0 (0%)	0.67
Pre-existing hypertension	21 (18%)	9 (28%)	0.5
Gestational diabetes	28 (24%)	3 (9%)	0.13
Gestational hypertension	15 (13%)	6 (19%)	0.03
*Delivery Outcomes*			
Gestational age at delivery (wk)	38.71 (2.9)	30.64 (8)	< 0.001
Birth weight (g)	2980 (12.1)	1270 (1387.5)	< 0.001
Apgar score 5 min	9 (2)	8 (2)	< 0.001
Mode of delivery: C-section	72 (61%)	26 (81%)	0.14
Preterm < 34 weeks	33 (28%)	28 (88%)	< 0.001
Small for Gestational Age (SGA)	10 (8%)	1 (3%)	0.31
Intrauterine growth restriction (IUGR)	27 (23%)	15 (47%)	0.01

Data are displayed as median (IQR) or n (%). Continuous variables were compared via Wilcoxon rank sum test, categorical variables were compared using χ^2^ test

*BMI* body mass index, *kg* kilogram, *PE* preeclampsia, *wk* week, *yr* year

### Biomarker performance

**Fig. 1:**
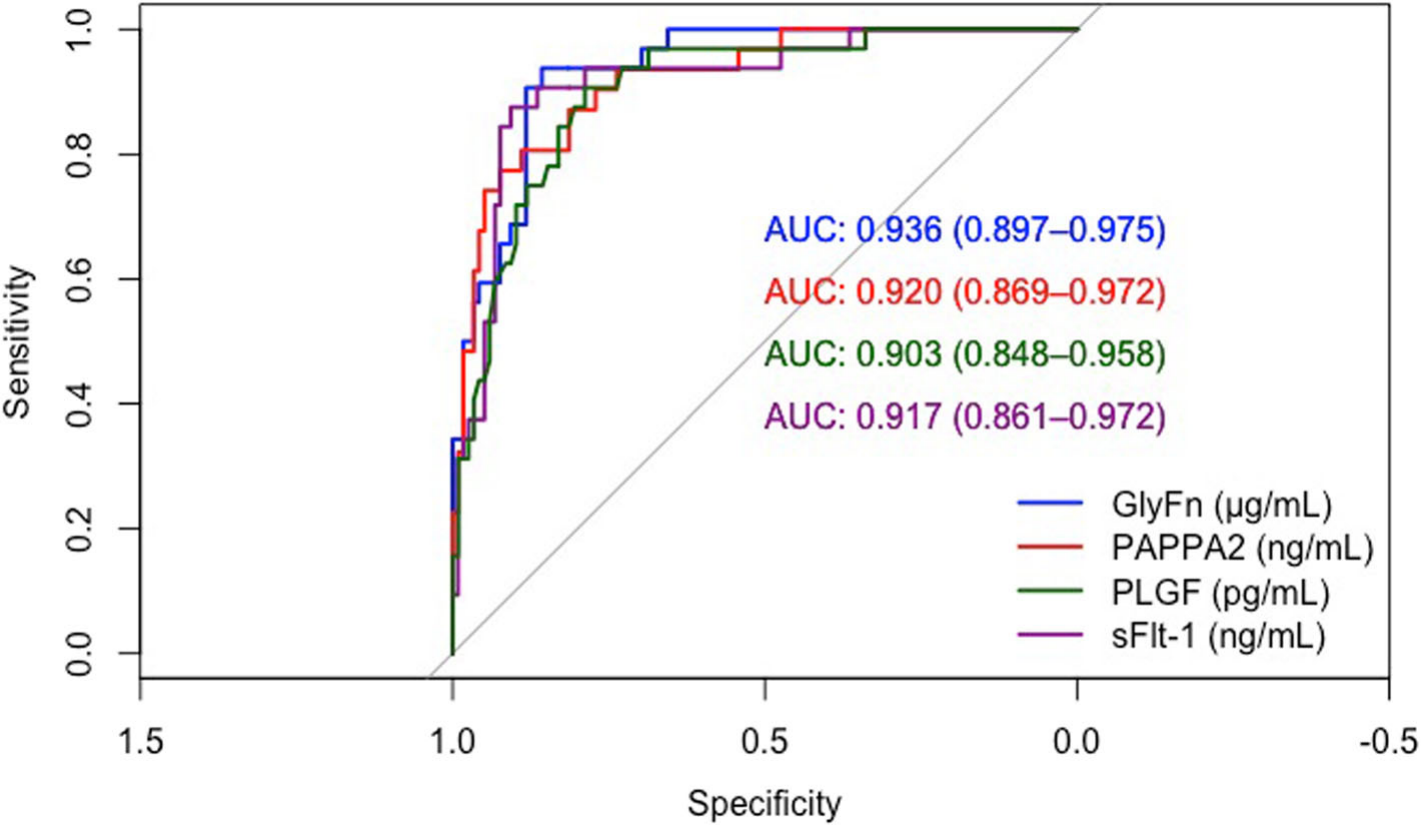
Receiver-operating characteristic curves showing the classification performance for each biomarker. AUC, area under the curve; GlyFn, glycosylated fibronectin; PAPPA2, pregnancy-associated plasma protein A2; PlGF, placental growth factor; sFlt-1, soluble fms-like tyrosine kinase-1

All analytes exhibited concentration differences between groups as shown in Table 2. The performance characteristics for prediction of PE within 4 weeks for all biomarkers are shown in Table 3. All biomarkers tested exhibited a high performance to rule-in or rule-out PE within 4 weeks of sampling [GlyFn AUC = 0.94 (95% CI, 0.90–0.97), PAPPA2 0.92 (95% CI, 0·88–0·96), PlGF 0.90 (95% CI, 0.84–0.95), and sFlt-1 0.93 (95% CI, 0.88–0.97)]. Figure 1 shows the ROC curves and associated AUCs of the biomarkers.

**Table 2:** Biomarker serum levels

	At-risk women without PE	At-risk women with PE	*p*-value
*n*	119	32	
Biomarker Levels			
GlyFn (μg/mL)	233 (92)	457 (206)	< 0.001
PAPPA2 (ng/mL)	72 (130)	505 (330)	< 0.001
PlGF (pg/mL)	275 (308)	40 (57)	< 0.001
sFlt-1 (ng/mL)	2186 (2170)	9869 (6613)	< 0.001

Data are displayed as median (IQR). Biomarker distributions are compared via Wilcoxon rank sum test

*GlyFn* glycosylated fibronectin, *PAPPA2* pregnancy-associated plasma protein-A2, *PlGF* placental growth factor, *sFlt-1* soluble fms-like tyrosine kinase-1

**Table 3:** Performance characteristics of biomarkers for short-term prediction of PE

Biomarker	AUC (95% CI)	Threshold	Sensitivity	Specificity
GlyFn (μg/mL)	0.94 (0.90–0.98)	315	91%	86%
PAPP-A2 (ng/mL)	0.92 (0.87–0.97)	200	87%	77%
PlGF (pg/mL)	0.90 (0.85–0.96)	100	81%	83%
sFlt-1 (ng/mL)	0.92 (0.86–0.97)	7000	84%	91%

*GlyFn* glycosylated fibronectin, *PAPPA2* pregnancy-associated plasma protein-A2, *PlGF* placental growth factor, *sFlt-1* soluble fms-like tyrosine kinase-1

### Performance of the Lumella™ POC test

**Fig. 2:**
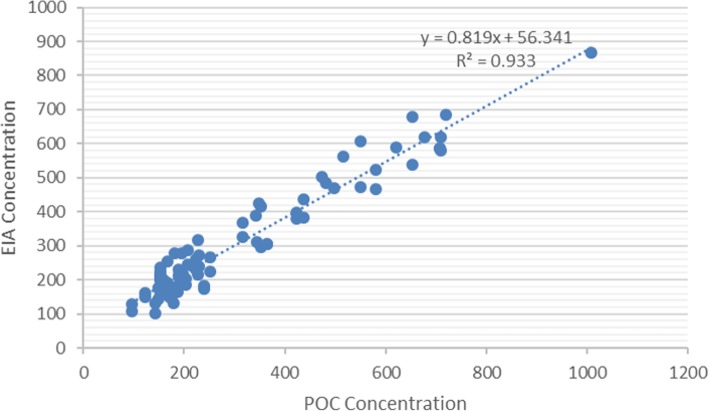
Correlation between GlyFn plate-based immunoassay (EIA) and Lumella™ POC test

The GlyFn plate immunoassay and the Lumella™ POC test were compared with a subset of randomly selected samples (*n* = 25 controls and 25 cases) that ranged from 100 to 900 μg/mL (the dynamic range of the Lumella™ reader system). There was a correlation of r = 0.966 between the two assay formats (Fig. 2). The ROC curves generated for both methods were in similar range between the plate (AUC = 0.94, 95% CI = 0.90–0·97) and POC (AUC = 0.99, 95% CI = 0.96–1.0) assays.

## Discussion

### Principal findings

The continuing revision of guidelines for prediction of PE [25] reflects the variation in clinical presentation, which makes accurate diagnosis based on a set of maternal signs and symptoms difficult in many cases. This uncertainty has focused attention in the last several years on maternal serum biomarkers as a potentially more consistent parameter for determining disease risk [26–30].Since 2010, the central focus of biomarker research has been on the diagnostic accuracy of commercially available immunoassays of the anti-angiogenic factor sFlt-1 and the pro-angiogenic factor PlGF and the sFlt-1/PlGF ratio. This study focused on the predictive value of a collection of previously described biomarkers in a large prospective observational cohort.

The biomarkers tested, GlyFn, PAPPA2, PlGF, and sFlt-1, all displayed good diagnostic performance for short-term (within 4 weeks) prediction of PE (AUROC of 0.90–0.94). Recent studies have focused on the investigation of pregnancies with signs and symptoms suggestive of PE, with the aim of identifying the development of PE within the subsequent 1–4 weeks. The Prediction of Short-Term Outcome in Pregnant Women with Suspected Preeclampsia study (PROGNOSIS) demonstrated that an sFlt-1/PlGF ratio < 38 exhibited a good NPV of 99.3% to rule out PE or HELLP within 1 week and that a ratio > 38 exhibited a PPV of 36.7% to rule in PE within 4 weeks [31]. Another prospective multicenter study reported an AUC of 0.87 for PlGF <5th percentile for the prediction of PE within 2 weeks [32]. The addition of systolic and diastolic blood pressure, uric acid, or alanine transaminase did not improve the diagnostic accuracy of PlGF alone. In comparison, GlyFn exhibited the best performance of the biomarkers tested in this study for prediction of PE within 4 weeks, with an AUC of 0.94, sensitivity of 91%, specificity of 86%. Additionally, the rapid GlyFn POC test, Lumella™, showed correlation of r = 0.966 with the standard plate assay in our study. The higher correlation and the AUC (0.99) for the Lumella™ assay is an improvement over these values for the earlier prototype (0.76 and 0.78, respectively) [18]. The GlyFn POC test may be of significant clinical utility for triage and intervention in low-resource settings or when the clinical diagnosis should be accurately and timely confirmed or excluded.

### Strengths and weaknesses

This is the largest and the first prospective study to evaluate the recently identified biomarkers GlyFn and PAPPA2 and the previous biomarkers sFlt-1 and PlGF in the prediction of PE. We also describe an improved version of a POC test for GlyFn (Lumella™).

A potential weakness of this study is that the proposed thresholds for GlyFn, PAPPA2, PlGF, and sFlt-1 are only initial suggestions for the use of these biomarkers as a simple combined biomarker test. All biomarker levels may vary with gestational age [33] and ethnicity, and may depend on maternal weight, smoker status, fetal growth [34] and parity [35]. These simplified cut-off values should be validated in a different study population before the panel could be integrated into clinical practice.

Because of a limited sample size, we were not able to test the diagnostic accuracy of the biomarkers in pre-existing proteinuria without hypertension. However, recently published studies have shown that PE can be accurately assed in women with chronic renal disease or lupus nephritis using PlGF and sFlt-1 [36–38].

Additionally, the set of biomarkers evaluated at less than 37 weeks of gestation may be restricted to the subset of early-onset potential placental PE. Late-onset PE is more likely to have maternal predisposing risk factors like obesity, diabetes mellitus, hypertension, or metabolic syndrome and varying levels of placental dysfunction [39, 40]. The performance of these biomarkers in late-onset PE was not evaluated as part of this study but might be improved with addition of maternal characteristics.

## Conclusion

Our results demonstrate that multiple biomarkers exhibit high performance in prediction of PE in the short term, and that GlyFn is adaptable to a POC format, joining the previously described POC test for PlGF [41].Therefore, we share the opinion of other researchers [26–30] that biomarkers should be incorporated into the definition of placental PE. A revised definition may reduce maternal and fetal mortality and morbidity as well as unnecessary healthcare usage. Additionally, the development of the GlyFn POC test may enable the extension of accurate, rapid, and inexpensive prediction of PE. It will be important to validate the performance of the GlyFn POC test in low and middle-income country settings and to evaluate its potential for detection of PE in early pregnancy and after 37 weeks of gestation.

## Data Availability

The anonymised data supporting our results can be obtained on request to the corresponding author Dr. Huhn.

## Abbreviations

ACOG: American College of Obstetricians and Gynecologists
AUC: Area under the (receiver-operating) curve
BMI: Body mass index
BP: Blood pressure
CI: Confidence intervals
c-section: Caesarean section
DNA: Desoxyribonuclein acid
EIA: Enzyme immunoassay
GlyFn: Glycosylated fibronectin
HELLP: Hemolysis, elevated liver enzymes and low platelets
ISSHP: International Society for the Study of Hypertension in Pregnancy
IUGR: Intrauterine growth restriction
PAPPA2: Pregnancy-associated plasma protein-A2
PE: Preeclampsia
PlGF: Placental growth factor
POC: Point of care
PROGNOSIS: Pregnant Women with Suspected Preeclampsia study
ROC: Receiver-operating characteristic
SD: Standard deviation
sFlt-1: Soluble fms-like tyrosine kinase-1
SGA: Small for gestational age
VEGF: Vascular endothelial growth factor
